# Unusual Epidemic of Tyzzer’s Disease in Commercial Rabbit Breeders: Clinical, Pathological, and Therapeutic Observations

**DOI:** 10.3390/ani15192920

**Published:** 2025-10-08

**Authors:** Benedetta Cordioli, Manuel Garbuio, Luca Palazzolo, Francesco Dorigo, Luca Zandonà, Laura Viel, Claudia Zanardello, Luca Bano

**Affiliations:** 1 Microbiology and Veterinary Diagnostic Laboratory, Istituto Zooprofilattico Sperimentale delle Venezie, Vicolo Mazzini 4, 31020 Treviso, Italylzandona@izsvenezie.it (L.Z.); lviel@izsvenezie.it (L.V.); lbano@izsvenezie.it (L.B.); 2 Rabbit Sector Veterinary Practitioner, 36061 Bassano del Grappa, Vicenza, Italy; dorivet60@gmail.com; 3 Histopathology Laboratory, Istituto Zooprofilattico Sperimentale delle Venezie, Viale Università 10, 35020 Legnaro, Padua, Italy; czanardello@izsvenezie.it

**Keywords:** Tyzzer’s disease, rabbit does, *Clostridium piliforme*

## Abstract

**Simple Summary:**

Tyzzer’s disease is a bacterial infection caused by *Clostridium piliforme*. While described in various animal species, it remains an uncommon disease in commercial rabbitries today, particularly in breeders. The clinical presentation and gross pathology may be variable and mislead the diagnosis towards other more common bacterial infections. This case report aims to provide a comprehensive overview of a Tyzzer’s disease case affecting only does in a commercial rabbitry. To the authors’ knowledge, this is one of the first reports to cover the clinical presentation, gross pathology, laboratory diagnosis, histopathology, and treatment of the disease affecting only adult female breeders.

**Abstract:**

*Clostridium piliforme* (Cp) is a pleomorphic spore-forming obligate intracellular bacterium and the causative agent of Tyzzer’s disease. The condition affects multiple species, including rabbits, in which the disease is sporadic in recently weaned animals. This report details a case of disease caused by Cp observed exclusively in breeding rabbits of a commercial farm. The clinical manifestations were a higher mortality rate in does and late-gestation abortions. We performed necropsy and further microbiological, parasitological and histopathological analyses. Anatomopathological lesions were suggestive of Tyzzer’s disease and the presence of Cp was confirmed by PCR. Parasitological analysis tested negative and standard bacteriological examination of intestines revealed a high load of *Escherichia coli* and *Clostridium perfringens*, which were considered secondary pathogens. *Chlamydophila* sp. and *Toxoplasma gondii* infections were excluded by PCR as causative agents of abortions. Moreover, in the months following the diagnosed outbreak, episodes of subcutaneous edema occurred in multiple does and young breeders born after the resolution of the epidemic. The constant reduction in the use of antimicrobials in recent years could make some neglected diseases emerge again. Therefore, it is crucial to suspect such uncommon pathologies in commercial rabbitries to properly manage them on farms.

## 1. Introduction

Ernst Tyzzer first described the disease in 1917 in a case of fatal diarrhea in a group of Japanese waltzing mice. He observed miliary necrotic foci of the liver and supposed the causative agent might be the pleomorphic, Gram-negative, motile, spore-forming bacillus he saw in cells bordering the areas of necrosis [1]. After 16S RNA sequencing, the obligate intracellular bacterium was classified as *Clostridium piliforme* (Cp) [2].

Ever since, the disease has been reported in multiple animal species such as cottontail rabbits, rats, hamsters, gerbils, guinea pigs, rhesus monkeys, foals, dogs, kittens and calves [3]. In rabbits, the infection spreads via the oro-fecal route and the clinical picture is characterized by anorexia, profuse watery diarrhea and mortality, especially in recently weaned rabbits [1,4]. The diagnosis can be presumed during necropsy in case of multiple tiny (1–2 mm diameter) necrotic foci on liver, together with edema and fibrotic plaque formation in the ileum, cecum and proximal part of the colon mucosa [5,6,7].

Presumptive diagnosis requires confirmation through the observation of the pathogen in histological slides (intracytoplasmatic long bacilli in silver-stained sections) and/or by its detection via biomolecular techniques. Cp cultivation is not routinely performed in veterinary microbiology laboratories because of the impossibility of growing the pathogen in standard media. Indeed, Cp can be isolated only in cell cultures and embryonated eggs [6].

Effective disease control requires early diagnosis, rapid removal of sick animals, prolonged antibiotic treatments and a proper cleaning-and-disinfection program [7].

To date, disease caused by Cp has been rarely reported and the epidemiology poorly investigated in commercial rabbitries. In the present report, we describe an epidemic field-case of Tyzzer’s disease observed exclusively in gestating does of a commercial rabbitry, causing high mortality and increased abortion rates, with the aim to suggest possible diagnostic processes.

## 2. Materials and Methods

### 2.1. Case History and Clinical Findings

The affected commercial rabbitry was located in Treviso, North-eastern Italy, and it was organized into 4 barns: 1 house for 3.000 commercial hybrid does with forced ventilation and 3 plein-air barns for the fattening period. Pregnant and young does were housed in wire cages with droppings pits underneath and were fed ad libitum with commercial feed. The house was connected to the public water supply. Rabbits were weaned at approximately five weeks of age.

Starting from 20th December 2022, does started dying (6.5% mortality) with profuse diarrhea and the farmer reported multiple abortions 7–3 days before deliveries (10% of inseminated does). The clinical picture appeared in animals irrespective of the parity order. Mortality and abortions were registered daily by the farmer.

### 2.2. Post Mortem Examination

On December 30th, three does (1–3) that died spontaneously underwent necropsy in the Microbiology and Veterinary Diagnostic Laboratory of Treviso (IZSVe). The number and specific carcasses to be sent to the lab were chosen by the veterinary practitioner. The selection was based on the condition of the carcass, prioritizing the animals with fewer post-mortem alterations, as well as the available funds. Pathology examination was performed according to the standard protocol [8]. Pathological samples were collected to perform bacteriological, parasitological and histopathological examinations.

### 2.3. Microbiological and Parasitological Examinations

Intestinal contents were aseptically collected and streaked on Eosin Methylene Blue (EMB) and Perfringens Agar Base (PAB). Livers were similarly processed and streaked on Blood Agar (BA) and EMB Petri dishes. Plates were incubated for 24–48 h at 37 °C in aerobic (BA and EMB) and anaerobic (PAB) conditions. Bacteria species were identified by means of traditional microbiology techniques and by MALDI-TOF Mass Spectrometry (MALDI-TOF MS, Brucker, Germany). *Escherichia* (*E.*) *coli* biotypes were determined by fermentation of 5 carbohydrates using the scheme of Camguilhem and Milon (1989) [9]. In addition, smears of intestinal contents (1–3) were Gram-stained in order to evaluate the presence of bacteria *Clostridium* (*C.*) *spiroforme*. Samples of cecum walls and livers were tested by end-point PCR for the presence of Cp [10].

Samples of spleen (3) and uterus (1 and 3) were tested for *Toxoplasma gondii* and *Chlamydophila* sp. by specific PCR protocols [11,12].

Parasitological examination of does 1–3 was performed individually through the microscopic observation of fecal smears and, in a representative pooled sample, by means of both the McMaster egg counting technique and immunofluorescent essay for the detection of *Cryptosporidium* sp. and *Giardia* sp. [13].

### 2.4. Histopathology and Histochemistry

Samples of ceca (1, 3), kidney (1) and spleen (3) were immediately fixed in 10% neutral-buffered formalin for 48h and routinely processed for histological investigations. Sections were stained with hematoxylin and eosin (HE). Silver stain was also performed on cecal sections to detect intracytoplasmatic argyrophilic bacilli.

### 2.5. Therapy

There are no antibiotics registered for the disease and antimicrobial susceptibility testing has never been performed because of technical difficulties in culturing Cp. As a consequence, the therapy was set up with oxytetracycline based on the experience described by Peeters et al. (1985) and it started on 9 January 2023 in drinking water (20 mg/kg/day; 5 days) followed by 5 cycles of antibiotic treatment in feed (1200 ppm/q) [7].

## 3. Results

### 3.1. Case History and Clinical Findings

**Figure 1:**
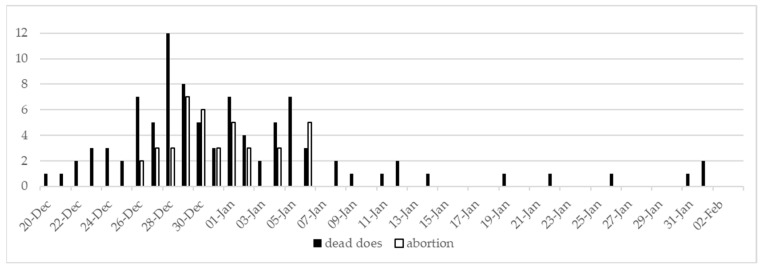
Number of dead does and abortions from December 20th to February 2nd in the affected farm.

The mortality and abortion trends are presented in Figure 1. The peak number of mortality and abortions in the affected barn was registered by the farmer between December 28th and 30th. From December 20th and January 9th, the overall mortality and total number of affected animals were 8.3% and 35.5%, respectively. The historical average mortality of the farm was 3‰.

### 3.2. Post-Mortem Examination

**Figure 2:**
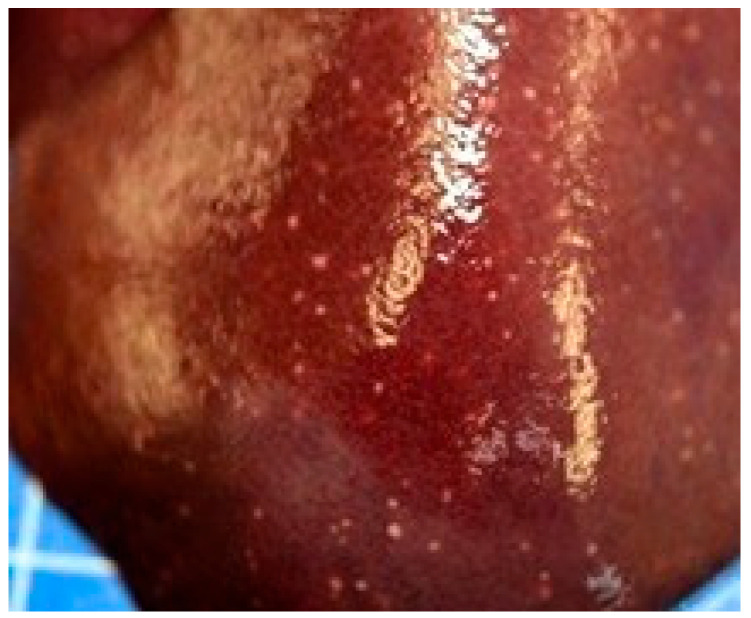
Doe 2. Multiple tiny foci on the liver surface, suggestive of necrosis.

Does 1–3 presented mild fecal staining of the perianal area. Does 1 and 2 had moderate serous effusion in the peritoneal cavity (approximately 5 mL) with multiple tiny (1–2 mm diameter) foci on the liver suggestive of necrosis (Figure 2).

**Figure 3:**
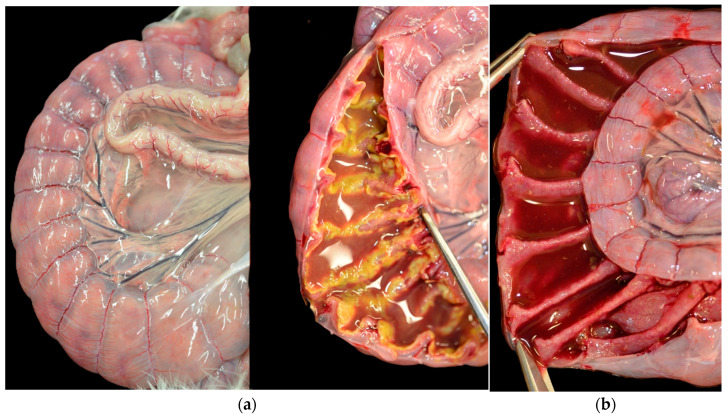
(**a**) Doe 1. Severe edema of the cecum wall (**left**) and yellow-gray necrosis in the cecal mucosa (**right**). (**b**) Doe 2. Fluid and hemorrhagic cecal content.

At the serosa of the cecum and large intestine mesentery, severe edema was a consistent feature in all of the animals. Doe 1 presented diffused areas of yellow-gray pseudomembrane strictly connected to the cecal mucosa, interpretable as necrosis. The others had fluid and hemorrhagic intestinal content, but the mucosa was not affected. Lymphadenopathy was not detected in any of the does (Figure 3).

Does 1 and 3 showed sub-capsular hemorrhagic petechiae on the kidneys’ surface and mild splenic enlargement, respectively. The uteri had necrotic placenta fragments and blood clots in the lumen.

### 3.3. Microbiological and Parasitological Examination

The results of the bacteriological and parasitological examinations are summarized in Table 1.

The bacteriological examination of the livers resulted negative.

All of the cecal walls (1–3) and the liver of doe 2 resulted positive for Cp, whereas all the other samples tested were negative.

In does 1–3, the testing for intestinal parasites resulted negative. Additionally, the targeted PCR essays for *Toxoplasma gondii* and *Chlamydophila* sp. were also negative.

### 3.4. Histopathology

**Figure 4:**
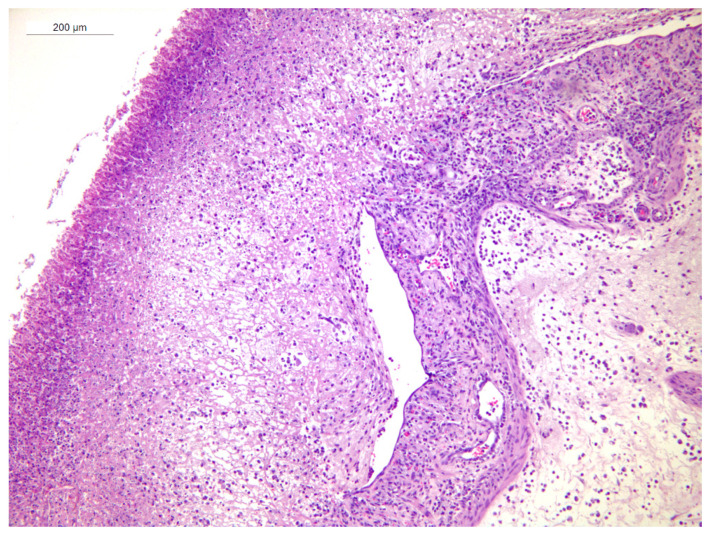
Doe 1, cecal wall. Severe, diffuse, fibrino-necrotic heterophilic typhlitis (HE stain).

In animal 1, multifocal to diffuse fibrino-necrotic heterophilic typhlitis with areas of erosion of the mucosa was observed. The lamina propria and the submucosa showed diffuse edema with moderate scattered infiltrates of heterophils and macrophages and fewer lymphocytes and plasma cells. Fibrino-necrotic material, heterophils, and cellular debris were present within the organ’s lumen and adherent to the mucosal surface (Figure 4).

**Figure 5:**
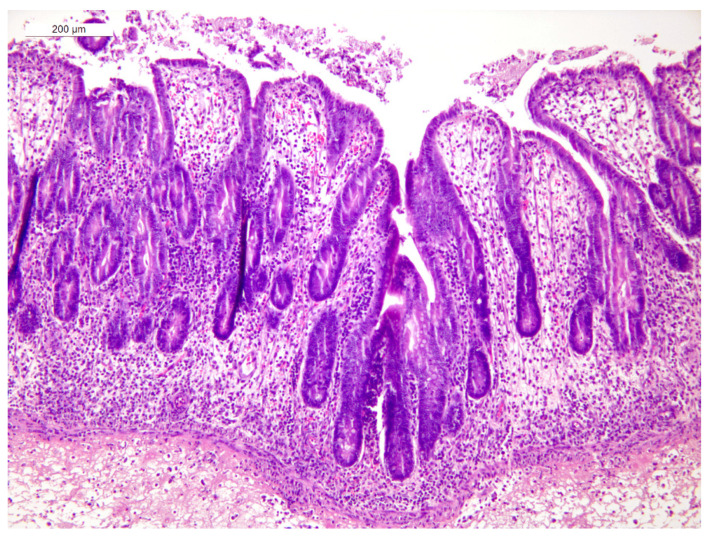
Doe 3, cecal wall. Edema and a moderate number of lymphocytes, plasma cells, heterophils and macrophages in the lamina propria and submucosa of the cecum. Foci of necrosis in the muscularis mucosae with mild heterophilic infiltrates are also visible (HE stain).

Animal 3 showed multifocal and moderate fibrino-necrotic heterophilic typhlitis with foci of erosion of the mucosa. The lamina propria and submucosa were diffusely edematous, with a moderate number of lymphocytes, plasma cells, heterophils and macrophages. Foci of necrosis of the muscularis mucosae with mild heterophilic infiltrates were also observed (Figure 5).

Fibrino-necrotic material, heterophils, and cellular debris were present within the organ’s lumen and adherent to the cecal mucosal surface

**Figure 6:**
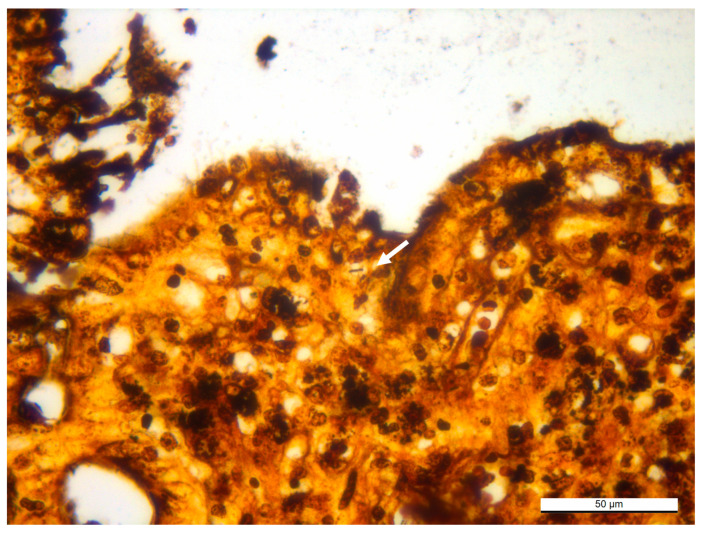
Doe 1, cecal wall. The white arrow points to the rare intracytoplasmic bacilli consistent with *C. piliforme* in the cytoplasm of the cecal epithelium (WS stain).

The Warthin–Starry (WS) stain showed the presence of rare argyrophilic intracytoplasmic bacilli consistent with *C. piliforme* in the cecal epithelium (Figure 6).

No significant histological lesions were observed in the kidney (1) and spleen (3).

## 4. Discussion

Tyzzer’s disease is a sporadic finding primarily reported in recently weaned rabbits. The present case report describes the first outbreak occurring only in breeders, to the best of the authors’ knowledge.

Although the clinical signs and anatomopathological lesions align with those documented in the literature, it is crucial to note that these findings, if considered individually, may potentially result in a misleading diagnosis. Indeed, differential diagnosis related to the disseminated point-like white lesions on the liver surface described in two out of three does might also be linked to other bacterial and parasitological diseases. Bacteriological examination of the livers ruled out the presence of *Salmonella* sp., *Staphylococcus aureus*, *Pasteurella multocida* and *Yersinia pseudotuberculosis* infections. Similarly, negative parasitological findings in the cecal contents made a hepatic infestation from *Eimeria stiedae* highly unlikely. Likewise, the intestinal lesions together with possible necrotizing lesions confined only to the liver led to ruling out *Francisella tularensis*. Indeed, this infection is common in wild rabbits and necrotic to granulomatous lesions involve both the liver and spleen [14].

Due to the consistent, uniform size of the necrotic lesions, *Cysticercus pisiformis* was excluded as an etiology. This condition is typically characterized by the migration of the *Taenia* (*T*.) *pisiformis* larval stage, leading to the development of fibrous tracts and necrotic foci [8]. The absence of contact with dogs, the definitive host of *T. pisiformis*, at this commercial farm also makes this parasitic disease very unlikely.

We ruled out also neoplastic causes for the liver lesions because the liver has never been reported in the literature as a primary site for tumors in rabbits. While hepatic lesions have been observed in a case of lymphosarcoma in a juvenile rabbit, those lesions were generalized and found in many other organs [15]. Additionally, the potential lymphoid nature of the hepatic lesions is difficult to link with the severe intestinal findings.

The severe edema of the cecal wall observed during necropsy could be the consequence of the action of the binary toxin produced by *C. spiroforme* [5,16]. However, the absence of bacteria with typical helicoid shape seen with the Gram staining of the intestinal contents, along with the absence of predisposing factors (long-term antibiotic treatment) led to ruling out a diagnosis of *C. spiroforme*-associated disease [17].

Long-term antibiotic treatment or oral administration of antibiotics considered toxic to rabbits (e.g., amoxicillin) are also known risk factors for *Clostridioides difficile* (CD) infection in humans and rabbits [18]. The lack of previous antimicrobial treatments supports a low probability of CD’s role in the intestinal lesions observed. In addition, CD in rabbit is rarely reported as a cause of typhlocolitis; as it mainly targets the small intestine and, contrarily to humans, in this species, pseudomembranous lesions associated with CD have never been described [18,19]. While the etiological role of CD in rabbit enteric disease remains to be investigated, a survey conducted in Italy on post-weaning rabbits revealed a low detection rate (approximately 3%). Furthermore, there was no statistically significant difference in CD detection between groups of rabbits affected and not affected by enteric disease [20].

Even though bacteriological examination of the intestines revealed the presence of *E. coli* B19 and B31 together with *C. perfringens*, histopathology did not show any Gram-negative bacteria attached to the luminal border of enterocytes, as described for some strains of enteropathogenic *E. coli* in rabbits, causing cecal edema and epithelial desquamation [21,22]. Therefore, such bacteria proliferation was considered a secondary reaction.

Even though some of the clinical signs could be attributable to other bacterial infections causing fetal deaths and stillbirths, primarily *Listeria* sp., the gross pathology of the uteri showed the consequences of recent abortions without any signs of bacterial infection. Indeed, does involved in a *Listeria monocytogenes* outbreak in a rabbit farm in Portugal showed major lesions on the reproductive apparatus, with thickening of the uterine wall and abundant purulent gray exudate, but not intestinal lesions, as we described [23]. Moreover, it was considered unlikely that testing only two out of three does would yield a false-negative result if the reproductive disease was caused by *Chlamydophila* sp. or *Toxoplasma gondii*. This assumption is based on the infectious nature of these agents and their high prevalence within an infected population, especially considering that the does shared the same feed, environment and management practices.

Considering the limitation of this case report due to the small number of does examined, we concluded that the enteric syndrome caused by Cp was the leading cause of both mortality and abortions (indirectly), because neither the lesions nor microbiological tests revealed the presence of any causative agent of reproductive disease. Direct bacteriological and/or histological examination of the uterus could have helped to confirm the diagnosis, even if no macroscopical abnormality was present.

The treatment started when mortality and abortions naturally decreased, impeding the evaluation of oxytetracycline’s efficacy in reducing the clinical manifestation in this outbreak of Tyzzer’s disease.

Remarkably, in the subsequent months (January–September 2023), 20 does exhibited diffuse subcutaneous and abdominal edema without enteric symptoms (pictures available in Supplementary Materials). This clinical manifestation was observed also in young breeders (75 days of age) born after the outbreak was controlled with antibiotics. In the following months and deliveries, the mortality rate did not increase, and the farmer did not report any abortions or reproductive problems. During 2023, eight does were humanly euthanized to perform further diagnostic analyses, but Cp DNA was never detected. However, this peculiar manifestation in a farm with a history of Tyzzer’s disease might be associated with this occurrence.

The ongoing reduction in antimicrobial use in the European Union could lead to the emergence of neglected and rare diseases like Tyzzer’s, even in breeding animals. For this reason, it is important to recognize the macroscopic findings and confirm the diagnosis as soon as possible by means of histopathological examinations and specific biomolecular analysis.

## 5. Conclusions

Tyzzer’s disease may affect breeders and diagnosis in rabbits is still rather difficult and might be misled if few animals are submitted for necropsy. Indeed, liver lesions are inconsistent and might be attributed to different pathogens and the intestinal ones are always present but may vary between subjects (from fluid hemorrhagic cecum contents to pseudomembranous typhlitis). Veterinarians and pathologists should be aware about the possibility of encountering the disease and the recognition of anatomopathological lesions followed by appropriate diagnostic examinations (PCR and histopathology) are necessary to diagnose Tyzzer’s disease.

## Figures and Tables

**Table 1:** Results of the diagnostic examinations.

Laboratory Examination	Sample	Method	Doe 1	Doe 2	Doe 3
Bacteriological examination	Intestinal content	Standard culture	*E. coli* B19 *C. perfringens*	*Citrobacter sediakii* *C. perfringens*	*E. coli* B31 *C. perfringens*
	Liver		Negative	Negative	Negative
Parasitological examination	Intestinal content	Direct microscopy	Negative	Negative	Negative
*C. spiroforme*	Intestinal content	Gram stain	Negative	Negative	Negative
*C. piliforme*	Cecal wall	PCR	Positive	Positive	Positive
	Liver		Negative	Positive	Negative
*Cryptosporidium* sp. and *Giardia* sp.	Pooled cecal content	Immunofluorescent essay	Negative		
*Toxoplasma gondii*	Spleen (doe 3) and uterus (does 1 and 3)	PCR	Negative	NT *	Negative
*Chlamydophila* sp.			Negative	NT *	Negative

* NT: not tested.

## Data Availability

No new data were created or analyzed in this study. The original contributions presented in this study are included in the article. Further inquiries can be directed to the corresponding authors.

## Supplementary Materials

The following supporting information can be downloaded at: https://www.mdpi.com/article/10.3390/ani15192920/s1, Figure S1: A 120-day-old rabbit doe presented with diffuse subcutaneous edema, which was both liquid and gelatinous. The edema was located primarily in the lower-back region, and to a lesser extent, in the ventral areas of the body. Figure S2: Abdominal serous effusion. Transparent, abundant effusions were also present in thoracic cavity and pericardium. In some cases, this was associated with rare, small, round, white foci on the liver and pale kidney parenchyma. Figure S3: Edematous caecal wall. This gross lesion was found in all rabbits with subcutaneous edema, despite specific PCR tests for Clostridium piliforme DNA on this organ consistently yielded negative results.
