# Supplementary material for: Unusual Epidemic of Tyzzer’s Disease in Commercial Rabbit Breeders: Clinical, Pathological, and Therapeutic Observations
Source: Animals (Basel). 2025 Oct 8;15(19):2920. doi: 10.3390/ani15192920 (PMC12524181; doi:10.3390/ani15192920)
Supplement: Supplementary file 1 [file animals-15-02920-s001.zip › animals-3843524-supplementary.pdf]

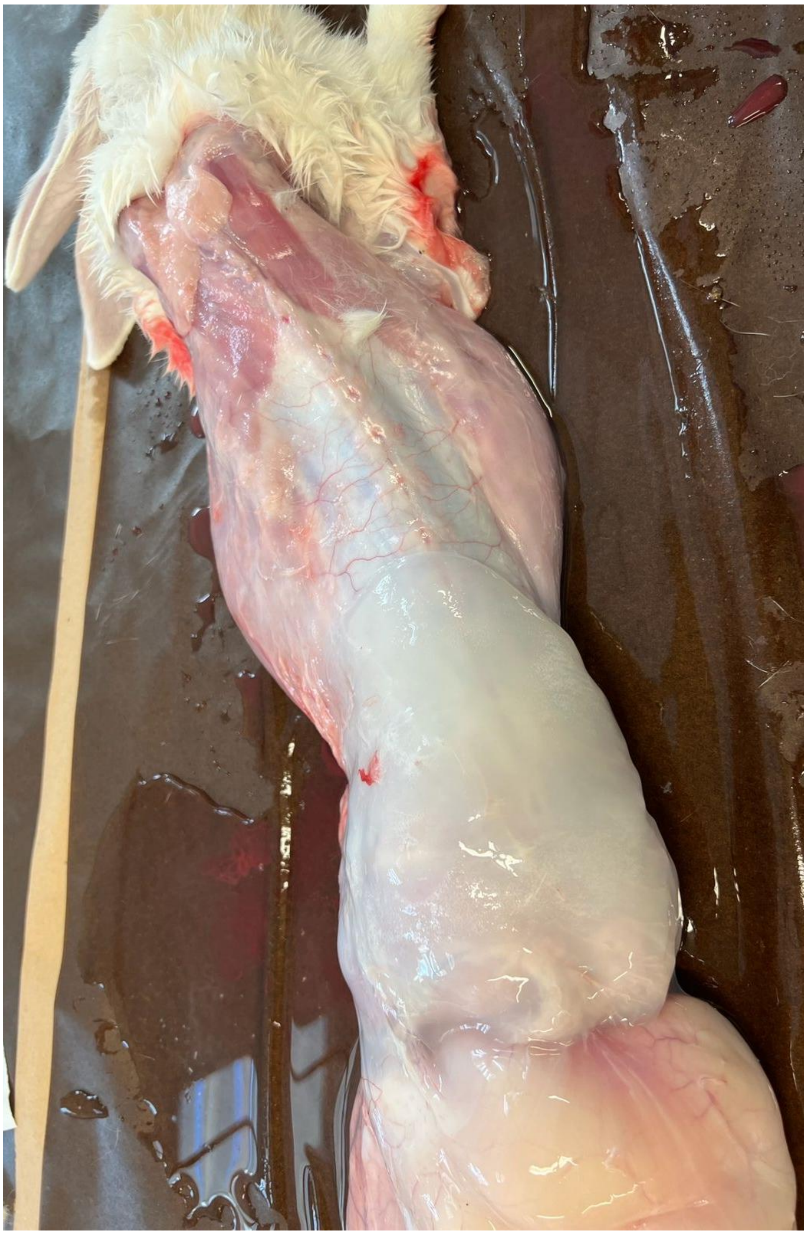

Figure S1. A 120-day-old rabbit doe presented with diffuse subcutaneous edema, which was both liquid and gelatinous. The edema was located primarily in the lower-back region, and to a lesser extent, in the ventral areas of the body.

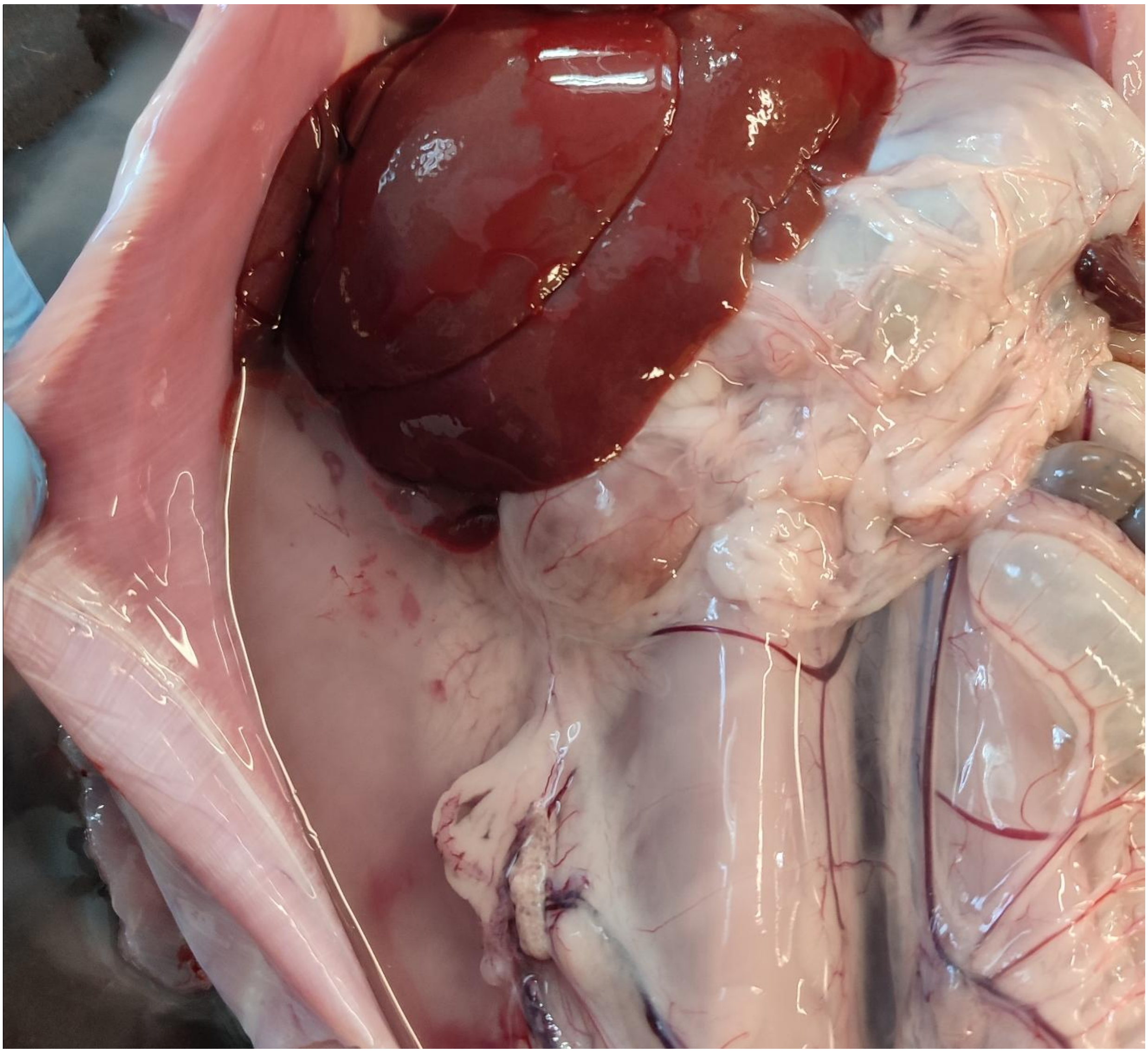

Figure S2. Abdominal serous effusion. Transparent, abundant effusions were also present in thoracic cavity and pericardium. In some cases, this was associated with rare, small, round, white foci on the liver and pale kidney parenchyma.

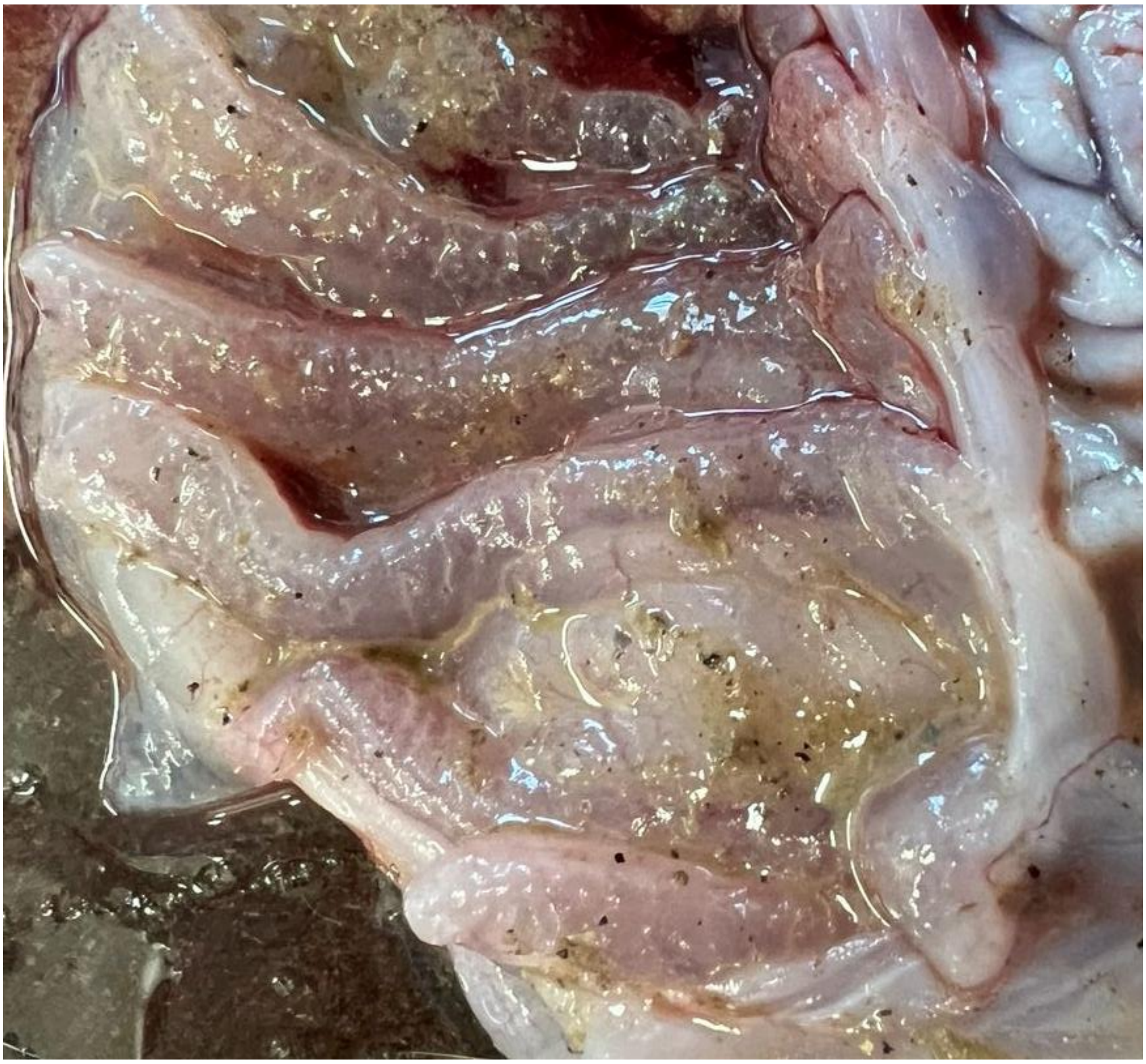

Figure S3. Edematous caecal wall. This gross lesion was found in all rabbits with subcutaneous edema, despite specific PCR tests for *Clostridium piliforme* DNA on this organ consistently yielded negative results.
